# Bioactive Dental Adhesive System With *tt*-Farnesol: Effects on Dental Biofilm and Bonding Properties

**DOI:** 10.3389/fbioe.2020.00865

**Published:** 2020-07-23

**Authors:** Diana Leyva del Rio, Neimar Sartori, Nichole Barton Tomblin, Jin-Ho Phark, Vanessa Pardi, Ramiro M. Murata, Sillas Duarte

**Affiliations:** ^1^Advanced Program in Operative and Adhesive Dentistry, Division of Restorative Sciences, Herman Ostrow School of Dentistry, University of Southern California, Los Angeles, CA, United States; ^2^Division of Periodontology Diagnostic Sciences, Dental Hygiene & Biomedical Science, Herman Ostrow School of Dentistry, University of Southern California, Los Angeles, CA, United States; ^3^Department of Foundational Sciences, School of Dental Medicine, East Carolina University, Greenville, NC, United States

**Keywords:** dental adhesive systems, *tt*-farnesol, universal adhesive, antibacterial activity, antibacterial adhesive system, hybrid layer, bond strength

## Abstract

**Background:**

Composite dental restorations are commonly used to restore cavitated carious lesions. Unfortunately, the main reason for failure is the development of secondary caries adjacent to the restoration. To improve the long-term survival of restorations, antibacterial agents have been added into dental materials. In this study, we assessed the antibacterial and bonding capacity of a commercial universal dental adhesive incorporated with the antibacterial agent *tt*-farnesol creating 3 experimental adhesives: 0.38% (v/v), 1.90% (v/v), and 3.80% (v/v), plus a control (no incorporation of *tt*-farnesol).

**Methods:**

The antibacterial activity was evaluated by assessing colony-forming units (CFU), biofilm dry weight (DW) and production of extracellular insoluble polysaccharides (EIP) at day 2, 3, and 5 of biofilm growth post surface treatment on the surface of composite disks. The effect of *tt*-farnesol on the chemical and bonding capacity of the adhesive system was assessed via pH analysis, degree of conversion (DC), and microtensile bond strengths to human dentin in both self-etch and etch-and-rinse application modes. A qualitative analysis of the effects of *tt*-farnesol on biofilm formation was evaluated using scanning electron microscopy (SEM). The sealing capacity of all adhesive systems tested was evaluated using confocal laser scanning microscopy (CLSM).

**Results:**

The 3.80% (v/v) experimental adhesive exhibited the lowest CFU count and lowest production of EIP at day 5. DW and pH values did no exhibit statistical differences among all tested groups. Bond strengths and DC decreased with the incorporation of the antibacterial agent into the adhesive system regardless of the concentration of *tt*-farnesol.

**Conclusion:**

The incorporation of *tt*-farnesol into the adhesive system significantly reduced bacterial viability and production of EIP; however, the bonding properties of the experimental dental adhesives were altered.

## Introduction

Cavitated dental caries lesions are usually treated by removing the infected tooth tissue and restoring the missing portion of the tooth with restorative materials such as adhesive composite restorations. The use of composite resin restorations has been increasing due to its high esthetics when compared to amalgam, however, in the long-term one of the main reasons for failure of these restorations is due to the occurrence of secondary caries adjacent to the restoration (Beck et al., 2015). Cariogenic biofilm plays an important role in the establishment and progression of secondary caries. In the oral microenvironment, biofilm forms due to complex interactions between microorganisms, sugar-rich diet, and the host that result in the production of acids that demineralize the dental substrate (Kidd and Fejerskov, 2004). Biofilms are microbial communities that are immersed in a three-dimensional extracellular matrix (EM) that have the capability of attaching to surfaces. From the wide range of microorganisms that are involved in the carious process, *Streptococcus mutans* is still considered the principal producer of EM in dental biofilms (Bowen and Koo, 2011). *S. mutans* is a highly aciduric and acidogenic microorganism that encodes glucosyltransferases (Gtfs) which produce extracellular polysaccharides in the presence of sucrose. Extracellular polysaccharides (insoluble and soluble) are the main constituent of the EM and have the capability of providing a supportive framework for biofilm development while promoting microbial adhesion to surfaces as well serving as a barrier to diffusion (Koo et al., 2013).

The formation of oral biofilms does not only occur on the surface of dental hard tissues, but also on the surface of restorative materials. Resin composites are in particular susceptibility to the initiation and development of biofilms because of its surface properties (Cazzaniga et al., 2015) affecting the longevity of the restoration in the oral cavity. A potential alternative for reducing the incidence of secondary caries is the incorporation of antibacterial agents into restorative materials (Chen et al., 2018). Amongst dental restorative materials, dental adhesive systems are the ideal material to have antibacterial properties due to their intimate contact with dental hard tissues. Although some highly acidic adhesives have been shown to produce some antibacterial effects against *S. mutans* (Imazato et al., 1998; Herrera et al., 2000), the low pH of these adhesive agents may cause bond degradation at the interface due the activation of matrix metalloproteinases (MMPs; Pashley et al., 2011) that ultimately reduces the life of the restoration. Therefore, to increase the long-term outcome of dental restorations, some antibacterial agents have been incorporated into commercial adhesive systems such as 0.12% chlorhexidine that is found in Peak Universal Bond and 5% of 12-methacryloyloxydodecypyridinium bromide (MDPB) that has been incorporated into Clearfil SE Protect. The limited number of adhesive systems with antibacterial properties currently in the market yields the need to investigate compounds that could potentially be added into dental adhesives.

Trans,*trans*-farnesol (*tt*-farnesol, 3, 7, 11 – trymethyl-2,6,10-dodecatrien-1-ol), is a natural sesquiterpene alcohol found in propolis which has demonstrated a reduction in the incidence and severity of dental caries with negligible effects on the oral microflora in a study *in vivo* (Koo et al., 2002a), demonstrating its potential as an antibacterial agent against cariogenic bacteria. The mechanism of action of *tt*-farnesol focuses on the disruption of the proton permeability of the *S. mutans* membrane, which alters the acid production, acid tolerance, and polysaccharide synthesis (Koo et al., 2002b, 2003; Jeon et al., 2011). Thus, the introduction of *tt*-farnesol into restorative materials is justified due its demonstrated antibacterial properties against cariogenic pathogens. In recent literature, *tt*-farnesol was incorporated into dental restorative materials such as a glass ionomer (de Castilho et al., 2019) and into a self-etch (5th generation) and etch-and-rinse (7th generation) dental adhesives (André et al., 2017). Although the addition of agents such as epigallocatechin-3-gallate have been recently introduced (Silva et al., 2020) into universal adhesive systems (8th generation) demonstrating an antimicrobial potential against *S. mutans*, the incorporation of *tt*-farnesol into universal adhesive systems has not yet been described in the literature. Universal adhesives are the latest addition to dental adhesive systems that have the advantage of a simplified less technique sensitive one-step bonding system that provides a stable bonded interphase to the dental substrate (Cuevas-Suárez et al., 2019) making it potentially beneficial to the integration of an antibacterial agent into this novel adhesive system.

The aim of this study was to investigate the antibacterial properties of experimental adhesives by incorporating different concentrations of *tt*-farnesol into a commercially available universal adhesive system and analyze its effect on *S. mutans* biofilm viability, quantified by colony forming units (CFU), biofilm dry weight (DW), and extracellular insoluble polysaccharide production (EIP). Furthermore, the physical and bonding properties of the experimental adhesives were assessed by pH of the solution, degree of conversion (DC), and microtensile bond strength (μTBS).

## Materials and Methods

### Antibacterial Properties of an Antibacterial-Incorporated Adhesive

#### Antimicrobial Activity

In order to measure the antimicrobial activity, *S. mutans* UA159 bacterial strain (ATCC# 700610) was used. An initial bacterial inoculum at 1 × 10^5^ was prepared with different concentrations of the *tt*-farnesol (3, 7, 11-trimethyl-2, 6, 10-dodecatrien-1ol; Sigma-Aldrich Co, St Louis, MO, United States) against *S. mutans* UA159, ranging from 10 to 500 μM of the antibacterial agent. The solutions were incubated at 37°C and 5% CO_2_ for 24 h. The minimum inhibitory concentration (MIC) of *tt*-farnesol was obtained by observing bacterial growth inhibition at 150 μM of the antibacterial agent and determined by triplicates.

#### Antibacterial-Incorporated Adhesive

A commercially available universal dental adhesive (Adper Scotchbond Universal, 3 M ESPE, St. Paul, MN, United States) was combined with the antibacterial agent *tt*-farnesol. Due to the liquid nature of the antibacterial agent, this was directly incorporated into the adhesive by pipetting for 90 s and then placed the mix in a centrifuge for 1 min at 1000 rpm to remove any air from the mix. Experimental adhesive systems at 3 different concentrations were fabricated based on the MIC: 100xMIC = 0.38% (v/v), 500xMIC = 1.90% (v/v), and 1000xMIC = 3.80% (v/v). The adhesive with no addition of *tt*-farnesol or any other solvent served as a control: 0xMIC.

#### Biofilm Preparation and Analysis

**FIGURE 1:**
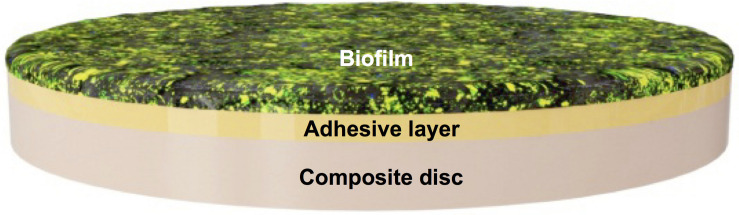
Representative illustration of *S. mutans* biofilm growing on top of a composite disk.

Composite disks of 13 mm in diameter and 1 mm thickness were fabricated with a microhybrid resin composite (Filtek Z250, 3 M ESPE, St. Paul, MN, United States) using a circular stainless steel mold to obtain smooth and uniform surfaces. The composite disks were sterilized with ethylene oxide and later degasified for 48 h. To evaluate the *S. mutans* UA159 strain biofilm growth on the composite disks, 20 μL (one drop) of the experimental adhesives was applied to one surface of the composite disks. The adhesive system was agitated on the surface of the disk for 20 s with a microbrush, air-dried, and light-cured for 10 s with a halogen unit (Elipar 2500, 3 M ESPE) with 600 mW/cm^2^ light output. The disks were then immediately placed in 24-well plates fully covered in 1 mL of BHI + sucrose + *S. mutans* (1 × 10^5^) inoculum medium for biofilm growth (Figure 1). The media was changed daily without disrupting the biofilm on top of the disks. Biofilm was collected at days 2, 3, and 5 after surface treatment and later sonicated in 5 mL of phosphate-buffered saline (PBS) solution to obtain a homogenized solution (Murata et al., 2008; Salles Branco-De-Almeida et al., 2011; Zancopé et al., 2016).

#### Colony Forming Units

Colony forming units’ assay was performed as followed: 1 mL of the homogenized solution was subjected to serial dilution (1 × 10^–1^–1 × 10^–4^), in which 20 μL of each dilution was inoculated in a tryptic soy agar plate with 5% sheep’s blood (TSA 5% SB) and placed in an incubator for 24–48 h at 37°C and 5% CO_2_ to allow colony growth. After this period, the colonies were counted and recorded. The numbers of colonies were normalized and converted into Log_10_ values for data interpretation. The CFU assay was performed in triplicate in at least three different experiments (Murata et al., 2008; Salles Branco-De-Almeida et al., 2011; Zancopé et al., 2016).

#### Biofilm Dry Weight

Two milliliters of the original homogenized suspension was completely dried in a SpeedVac concentrator (Thermo Scientific, Rockford, IL, United States) to obtain a dry pellet. The dry pellet was first weighted to obtain the biofilm biomass DW and later processed for EIP assay (Murata et al., 2008; Salles Branco-De-Almeida et al., 2011; Zancopé et al., 2016).

#### Production of Extracellular Insoluble Polysaccharides

After obtaining and weighting the pellet for the biofilm DW analysis, the pellet was resuspended and washed in 1 ml of water; this procedure was repeated twice to remove the soluble polysaccharides. The pellet was dried in a SpeedVac concentrator and the extracellular insoluble (alkali soluble) polysaccharides were extracted using 0.05 mL of 1 M NaOH per 1 mg of biofilm biomass DW. The samples were homogenized for 1 min, maintained under agitation for 3 h at room temperature and the concentration of insoluble carbohydrate was determined in the supernatant by a phenol–sulfuric acid method (Dubois et al., 1956); determination of polysaccharides by colorimetric assays has been widely used to estimate the polysaccharide content in dental plaque and *S. mutans* biofilms (Almeida et al., 2008; Murata et al., 2008, 2010; Zancopé et al., 2016).

#### Scanning Electron Microscopy Analysis

Disks with 5-day grown biofilms were processed for scanning electron microscopy (SEM) analysis. The specimens were immersed in 2.5% glutaraldehyde in 0.1 M sodium cacodylate buffer (Electron Microscopy Sciences, Hatfield, PA, United States) at pH 7.4 for 12 h at 4°C, rinsed with 0.2 M sodium cacodylate buffer (Electron Microscopy Sciences, Hatfield) at pH 7.4 for 1 h for three periods, followed by a distilled water rinse for 1 min. The specimens were then dehydrated in ascending concentrations of ethanol (25% for 20 min, 50% for 20 min, 75% for 20 min, 95% for 30 min, and 100% for 60 min). Subsequently, the specimens were immersed in hexamethyldisilazane (Electron Microscope Sciences, Fort Washington, PN, United States) for 10 min and then placed on a filter paper inside a covered glass vial and air-dried at room temperature inside a fume hood for 12 h (Dekker et al., 1991). The dried specimens were mounted on aluminum stubs and sputter-coated with gold/palladium. Images of specimens were obtained using a field-emission scanning electron microscope (JSM-7001F, JEOL, Tokyo, Japan) at an accelerating voltage of 5–0 kV with up to 10,000 times of magnification.

### Chemical Properties of an Antibacterial-Incorporated Adhesive

#### pH Measurement

The pH values of the control and each of the experimental groups were measured with a pH meter (Mettler Toledo, Columbus, OH, United States). The meter probe was calibrated with a buffer standard solution then submerged in 2 mL of the experimental solution for 2 min. For each measurement, pH values were recorded and the tip of the probe was rinsed with ethyl alcohol and distilled water to remove any remnants of previous solution. Measurements were performed under minimum light to avoid polymerization of the adhesive systems. Five different readings for each individual adhesive were performed, and the mean pH value was calculated.

#### Degree of Conversion

The DC was evaluated using micro-Raman spectroscopy (InVia, Spectrometer, Renishaw, New Mills, United Kingdom) with a laser wavelength of 532 nm, power output at 750 mW, microscope objective of 50X, and with a pin-hole aperture of 370 μm. The spectra range obtained was from 400 to 2000 cm^–1^, with an integration time of 20 s using a RenCam CCD detector with a 1024 × 256 pixel resolution. Data was processed using Origin2015 software (OriginLab Corporation, Northampton, MA, United States).

Three different samples were fabricated for the control and each of the experimental adhesives. After spectrometer calibration with a silicon sample, one drop (20 μL) of *tt*-farnesol-adhesive mixture was placed on top of a glass slide and spectra of the uncured adhesive was obtained and recorded. The adhesive drop was agitated in top of the sample for 20 s with a microbrush and then gently air thinned for 5 s to promote solvent evaporation. A thin glass slide was placed on top of the drop to both create a uniform layer and prevent oxygen inhibition layer formation. The surface was light-cured for 10 s. The thin glass slide was removed and additional spectra of the cured sample was taken and recorded. At least 5 spectra per sample were recorded. The DC was calculated using the formula, $D\,C\%=\left[1-\frac{R\,c\,u\,r\,e\,d}{R\,u\,n\,c\,u\,r\,e\,d}\times 100\right]$ in which R is the ratio of areas under the aliphatic 1639 cm^–1^ peak and aromatic 1609 cm^–1^ peak in cured and uncured material.

### Bonding and Sealing Properties of an Antibacterial-Incorporated Adhesive

#### Microtensile Bond Strength

Caries-free human third molars where collected in accordance of the University of Southern California’s IRB (IRB #APP-13-05027). The external surface of the teeth was cleaned of any remaining organic debris and stored in 0.5% chloramine-T solution for no more than 2 months prior to μTBS testing and hybrid layer permeability analysis. 24 teeth were sectioned perpendicular to the longitudinal axis of the tooth, 2.5 mm above the cement-enamel junction (CEJ), and 3 mm bellow the CEJ using a low speed diamond saw (Isomet 1000, Buehler Ltd., Lake Bluff, IL, United States) and water-cooling to remove the coronal enamel and root. The occlusal surfaces of the crown segments were polished using a waterproof 600-grit silicon carbide paper under running water for 60 s to create a standard smear layer (Pashley, 1984). The crown segments were sorted into eight experimental groups using two bonding approaches, etch-and-rinse mode and self-etch mode for each of the experimental groups. The adhesive system was applied following the manufacturers’ instructions (Table 1) and light-cured for 10 s. Microhybrid composite resin (Filtek Z250, 3 M ESPE) was built up on top of the bonded surfaces with 3 successive increments of 2 mm, each light-cured for 20 s.

**TABLE 1:** Adhesive system composition and its application, according to the manufacturer recommendations.

**Adhesive**	**Composition**	**Application modes**
Adper Scotchbond Universal (3 M ESPE)	10-MDP Phosphate monomer Dimethacrylate resins HEMA Methacrylate-modified polyalkenoic acid copolymer Fillers Ethanol Water Initiators Silane	*A. Two-step etch-and-rinse* Apply etchant for 15 s Rinse Blot dry Apply adhesive for 20 s rubbing it against the tooth surface Air dry for 5 s until the adhesive doesn’t move Light-cure for 10 s
		*B. One-step self-etch* Apply adhesive for 20 s rubbing it against the surface Air dry for 5 s until the adhesive doesn’t move Light-cure for 10 s

After 24 h of storage in distilled water at 37°C, the samples were cut under water-cooling in both X/Y axis parallel of the long axis of the tooth to obtain untrimmed sticks with a cross-sectional surface area of 0.8 ± 0.2 mm^2^. Specimens were measured with a digital caliper (Mitutoyo digital caliper, Mitutoyo Corp., Tokyo, Japan) to obtain the bonded surface area of the interface and these values were recorded. Later, specimens were fixed to a testing jig with cyanoacrylate glue (Zapit, Dental Ventures of America, Corona, CA, United States), placed in a universal testing machine (Instron Model 4400, Instron Corp., Canton, MA, United States), and loaded in tensile force at a cross-head speed of 1 mm/min until fracture. The load (Newtons) and the bonding surface area (mm^2^) of the specimens were registered using the software TestWorks 4 (MTS Nano Instruments, Eden Prairie, MN, United States). μTBS were calculated by dividing the maximum load (N) applied in tension before failure by the bonding surface area (mm^2^) to obtain Mega Pascals (MPa) values. The failure modes were evaluated at 32× of magnification in a stereoscopic microscope and classified as cohesive (failure entirely within dentin substrate or resin composite), mixed (failure at dentin/resin interface including cohesive failure of one of the substrates), or adhesive (failure at the dentin/resin interface).

#### Hybrid Layer Permeability

Two additional teeth from each group were prepared for confocal laser scanning microscopy (CLSM) to observe the interaction between the adhesive system and internal dentinal fluids at the dentin-resin layer. Three drops of each adhesive system were mixed with one grain of tetramethylrhodamine B isothiocyanate (Sigma-Aldrich) to form a homogeneous solution. Tooth samples were restored using the methodology described above for each of the groups. After 24 h of storage in water, the pulpal chambers were filled with a solution of 0.1% fluorescein sodium (Sigma-Aldrich) for 4 h. The restored tooth segments were cut perpendicular to the adhesive interface using a slow-speed precision saw to obtain 2–3 slabs with 1.0 mm thickness from the center of the tooth. The samples were evaluated under a CLSM (LSM 5 Pascal, Zeiss, Jena, Germany) and images of the adhesive interface with 63 times of magnification at 1024 × 1024 pixels and 2.0 μs/pixel speed were obtained.

### Statistical Analysis

To detect differences between all the groups, data from at least 3 separate experiments were analyzed for the CFU, DW, and EIP assays. The three time points of evaluation were independently analyzed in each of the assays. This data was checked for normality with Shapiro–Wilk test. When normal distribution was detected, data was analyzed with one-way ANOVA followed by Tukey HSD *post-hoc* test (α = 0.05). When no normal distribution was detected, nonparametric multiple comparisons test with Dunn *post hoc*-test was used (α = 0.05). For μTBS tests, pH, and DC evaluation, data was checked for normality with Kolmogorov–Smirnov test. μTBS data was analyzed using two-way ANOVA followed by Tukey HSD post-hoc test (α = 0.05) to detect differences between both application modes in all the groups, meanwhile DC and pH values were analyzed using one-way ANOVA followed by Tukey HSD post-hoc test (α = 0.05).

## Results

### Antibacterial Properties of an Antibacterial-Incorporated Adhesive

#### Colony Forming Units

**FIGURE 2:**
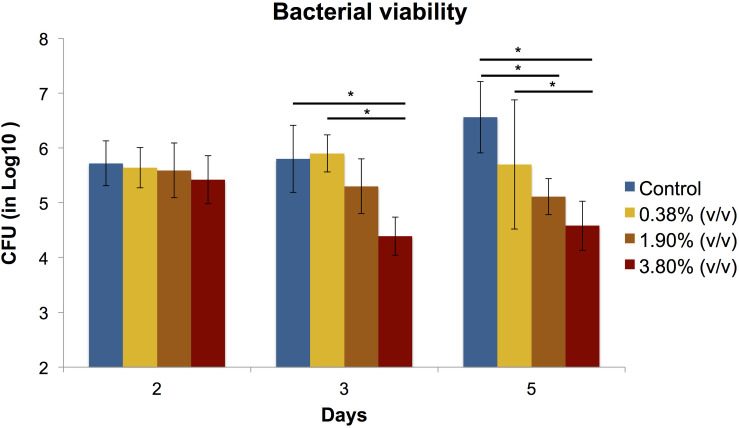
Means (±SD) of CFU (in Log10) count in *S. mutans* biofilms according to the experimental adhesives used at 2, 3, and 5 days after surface treatment. Groups connected by bars with an asterisk are statistically significant (*P* < 0.05).

The effects of the different concentrations of *tt*-farnesol on the CFU activity are shown in Figure 2. No statistical differences (*P* > 0.05) were found between groups at day 2. At day 3, results show statistical differences (*P* < 0.05) between control and 3.80% (v/v) groups and 0.38% (v/v) with 3.80% (v/v) groups. At day 5, differences between groups was highly evident showing a significant reduction in CFU values between control vs. 1.90% (v/v) and 3.80% (v/v) groups and it was also observed a significant difference between 0.38% (v/v) group and 3.80% (v/v) group.

#### Biofilm Dry Weight

**FIGURE 3:**
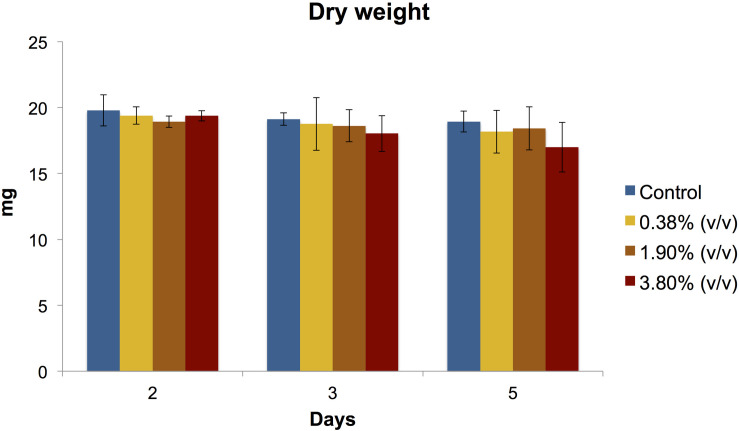
Means (±SD) of dry biomass weight (in mg) production of *S. mutans* biofilms according to the experimental adhesives used at 2, 3, and 5 days after surface treatment. There was no statistical difference (*P* > 0.05) between the experimental groups evaluated at any day.

There was no statistical difference (*P* > 0.05) in biofilm biomass among all tested groups at any time point evaluated (Figure 3).

#### Production of Extracellular Insoluble Polysaccharides

**FIGURE 4:**
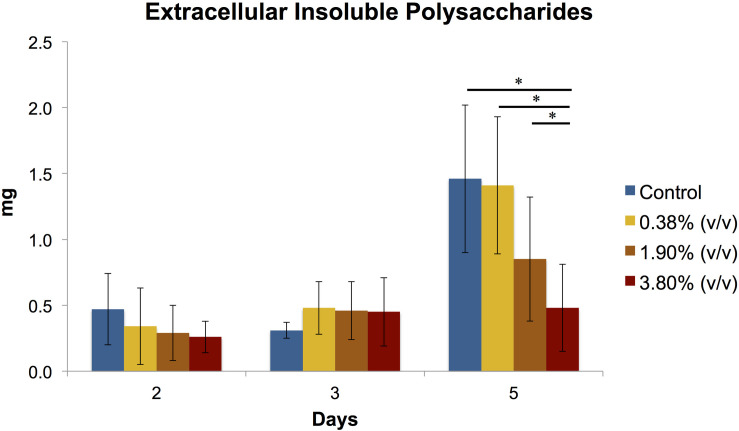
Means (±SD) of extracellular insoluble polysaccharides (in mg) production of *S. mutans* biofilms according to the experimental adhesives used at 2, 3, and 5 days after surface treatment. Groups connected by bars with an asterisk are statistically significant (*P* < 0.05).

At days 2 and 3, no statistical differences were observed amongst the groups (Figure 4). At day 5, it can be observed a 3-fold increase in the production of extracellular polysaccharides in both control and 0.38% (v/v) groups when compared to the previous time points evaluated. A significant difference (*P* < 0.05) in the amount of EIP production was observed for 3.80% (v/v) group and the rest of the groups.

#### Scanning Electron Microscopy

**FIGURE 5:**
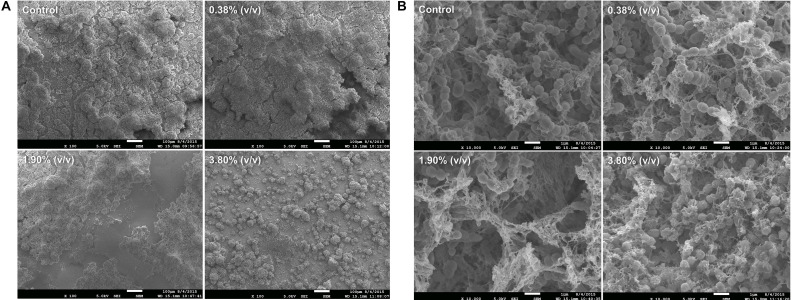
Representative SEM images revealing the biofilm growth on the top to the adhesive layer at 100× magnification **(A)** and at 10,000× magnification **(B)**. Control: 0.0% of *tt*-farnesol; 100×MIC: 0.38% (v/v) of *tt*-farnesol; 500×MIC: 1.90% (v/v) of *tt*-farnesol; and 1000×MIC: 3.80% (v/v) of *tt*-farnesol.

Scanning Electron Microscopy images exhibited bacterial colonies coating the surface of the composite disks. At 100× magnification (Figure 5A), it can be observed a thick and uniform biofilm formation covering the surface of the disk on both control and 0.38% (v/v) groups. On the other hand, for group 3.80% (v/v), it can be observed a disruption and reduction in biofilm formation showing individual bacterial colonies that spread throughout the surface of the disk. Higher magnification reveals chain-like cell arrangements of the *S. mutans* surrounded by fibrous hair-like structures of extracellular polysaccharides that are present in all groups (Figure 5B), on the other hand, on group 3.80% (v/v) it can be observed disorganized bacterial chain formation surrounded by a decreased and irregular EIP production.

### Chemical Properties of an Antibacterial-Incorporated Adhesive

#### pH Measurement

The pH mean values varied from 2.98 to 3.01 and there was no statistical difference (*P* > 0.05) amongst the experimental groups or the control group (Table 2). The addition of *tt*-farnesol did not alter the pH of the universal adhesive system Adper Scotchbond Universal.

**TABLE 2:** pH, degree of conversion (DC) in % and microtensile bond strength (μTBS) in MPa results.

**Group**	**pH**	**DC (%)**	**μTBS**	
			**Etch-and-rinse mode**	**Self-etch mode**
Control	2.978 ± 0.037^a^	73.11 ± 8.54^a^	74.34 ± 26.1^aA^	66.90 ± 16.4^aA^
0.38% (v/v)	3.010 ± 0.031^a^	49.23 ± 6.06^b^	64.29 ± 17.8^bA^	54.35 ± 20.9^bB^
1.90% (v/v)	2.990 ± 0.041^a^	48.78 ± 18.38^b^	59.80 ± 13.5^bA^	55.71 ± 19.9^bA^
3.80% (v/v)	2.994 ± 0.029^a^	45.48 ± 10.04^b^	55.69 ± 16.0^bA^	57.69 ± 19.1^bA^

*Means, standard deviation and statistical results of control and experimental adhesive systems bonded to human dentin in etch-and-rinse and self-etch modes. Within the same vertical column, means with same superscript lower-case letters (comparing different concentrations of *tt*-farnesol) are not statistically different (*P* > 0.05). Within the same horizontal row, means with the same superscript upper-case letters (comparing application mode) are not statistically different (*P* > 0.05).*

#### Degree of Conversion

The incorporation of the antibacterial agent decreased the DC of all experimental groups, regardless of its concentration of *tt*-farnesol. The control group obtained the highest mean with a 73.11% monomer-to-monomer conversion, showing a statistical difference (*P* < 0.05) from all the experimental groups. The mean DC values of the experimental groups ranged between 45.48 and 49.23% and no statistical differences were observed amongst the experimental groups (*P* > 0.05; Table 2).

### Bonding and Sealing Properties of an Antibacterial-Incorporated Adhesive

#### Microtensile Bond Strength

A reduction of μTBS was observed in all experimental groups (*P* < 0.05) when compared to the control group regardless of the application mode (Table 2). The control groups in both bonding modes yielded the highest bond strength means, 74.34 ± 26.1 MPa for the etch-and-rinse mode and 66.90 ± 16.4 MPa for the self-etch mode. When comparing etch-and-rinse and self-etch bonding modes, there were no statistical differences (*P* > 0.05) between the two in the control, 1.90% (v/v), and 3.80% (v/v) groups. Pre-testing failure occurred only in the experimental adhesives applied in the self-etch mode (Table 3). The percentage of adhesive failure increased when *tt*-farnesol was incorporated within the adhesive system, regardless of the application mode, indicating that *tt*-farnesol alters adhesive and dentin interaction.

**TABLE 3:** Failure mode distribution (%).

**Application mode and Experimental adhesives**	**Failure mode**				
	**PTF**	**A**	**CD**	**CC**	**M**
**Etch-and-rinse mode**					
Control	0	56.1	43.9	0	0
0.38% (v/v)	0	44.8	50	5.2	0
1.90% (v/v)	0	54.0	44.4	2.6	0
3.80% (v/v)	0	70.0	30.0	0	0
**Self-etch mode**					
Control	0	43.6	52.6	3.8	0
0.38% (v/v)	5.3	69.2	26.9	0	0
1.90% (v/v)	7	84.7	9.4	1.2	0
3.80% (v/v)	7	68.2	25.0	2.3	0

*Classification of failure modes: (PTF) Pre-testing failures: Specimens that failed before testing. (A) Adhesive: adhesive failure along bonded interface. (CD) Cohesive failure within dentin. (CC) Cohesive failure within composite. (M) Mixed failure: adhesive failure along bonded interface associated with a cohesive failure within dentin or composite.*

#### Permeability of the Hybrid Layer

**FIGURE 6:**
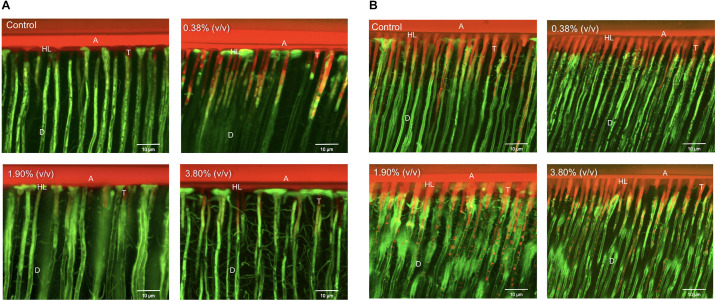
Representative CLSM images of the adhesive interfaces of the different experimental groups bonded using the etch-and-rinse **(A)** and self-etch **(B)** modes. Control: 0.0% of *tt*-farnesol; 100×MIC: 0.38% (v/v) of *tt*-farnesol; 500×MIC: 1.90% (v/v) of *tt*-farnesol; and 1000×MIC: 3.80% (v/v) of *tt*-farnesol. A: Adhesive layer; D: Dentin; T: Resin tags; HL: Hybrid layer.

Representative CLSM images illustrate the resin-dentin interface created by Adper Scotchbond Universal and the experimental adhesives when it was applied in both etch-and-rinse (Figure 6A) and self-etch (Figure 6B) modes. The adhesive resin is shown dyed in red, water dyed in green, whereas yellow areas represent the mixture between unpolymerized resin and water. For the etch-and-rinse groups, the hybrid layer formed in the control and 0.38% (v/v) groups adequately sealed the adhesive interface. The intradentinal hybrid layer around the resin tags obstructed water ingress from the pulp into the hybrid layer, thus preventing the hydrolytic degradation of the adhesive interface. However, when higher concentrations of *tt*-farnesol were incorporated into the adhesive system, monomers dissolved from the basal portion of the resin tags (shown as red globules inside dentin tubules). The resin tags formed on 1.90% (v/v) and 3.80% (v/v) groups were shorter than the control group, which did not allow for proper sealing the resin-dentin interface. For the self-etch approach, resin tags were shorter than those created by the etch-and-rinse approach. Water accumulation could be observed at the resin-adhesive interface in all experimental groups showing a different sealing capability of the experimental adhesive systems.

## Discussion

Dental caries is considered the most prevalent disease affecting populations worldwide which results in billions of individuals suffering from pain, loss of function, compromised esthetics, and speech (Kassebaum et al., 2015). Dependent on size and severity, carious lesions are generally treated by removing all demineralized and contaminated dental tissue and replacing it with a composite restoration. Ideally, these restorations would last a lifetime, reducing costs and discomfort to the patient. Unfortunately, a significant amount of restorations fail due to the initiation and progression of secondary caries adjacent to the restorative material. In order to prolong the longevity of composite restorations, antibacterial agents have been added into both experimental and commercial dental materials in an effort to counteract the activity of cariogenic bacteria and consequently reduce the incidence of secondary caries (Chen et al., 2018). Some antibacterial agents recommended for caries control such as chlorhexidine, and triclosan are based on their ability to reduce *S. mutans* viability, unfortunately, they exert a wide spectrum of antimicrobial activity also suppressing benign oral microflora. Thus, natural alternatives that specifically target important virulence factors like microbial adherence and polysaccharide synthesis (Seleem et al., 2016a, b) without altering normal microflora is an attractive therapeutic alternative (Philip et al., 2019).

In biofilms, microorganisms generally account for less than 10% of the dry mass, meanwhile the EM accounts for the remaining 90%. This matrix mostly consists of extracellular polysaccharides formed by the microorganisms themselves and in which they are embedded. These multifunctional extracellular polysaccharides will form a three-dimensional scaffold that is also responsible for the adhesion of the biofilm to surfaces, the aggregation of additional bacterial cells, providing mechanical stability of the biofilm and providing a protective barrier to specific host defenses (Flemming and Wingender, 2010). Therefore, it is crucial that novel antibacterial therapies target these important virulence factors of cariogenic bacteria to prevent the initiation and/or progression of dental caries. In this study, novel experimental dental adhesives effectively reduced virulence factors such as EIPs and viable cells of *S. mutans* biofilm demonstrating the potential for the delivery of an anticariogenic agent at the bonded interfaces.

We incorporated for the first time different concentrations of the natural antibacterial agent *tt*-farnesol into a commercially available universal adhesive forming experimental adhesives, which some significantly reduced both production of extracellular polysaccharides and cell viability of *S. mutans* biofilm. At day 2 and 3 after treatment, the effect of *tt*-farnesol on EP production had not yet manifested; however, at day 5 the experimental adhesive at 3.80% (v/v) concentration showed a significant reduction (*P* < 0.05) when compared to its control and was closely followed by the 1.90% (v/v) experimental adhesive. A similar trend could be observed for the *S. mutans* viability of the biofilms where a significant reduction (*P* < 0.05) of recoverable viable cells at day 3 was observed in the 3.80% (v/v) experimental adhesive when compared with the control. At day 5 this trend becomes more evident where this group shows a ≈2 log_10_ difference with the control group. Additionally, the 1.90% (v/v) experimental adhesive shows a ≈1.5 log_10_ difference when compared also to the control group. Mean biomass DW values obtained from all experimental adhesives were lower than its control group at the three time points evaluated, however, these values were not statistically significant (*P* > 0.05). This study is consistent in demonstrating the antibacterial activity of the propolis-derived agent *tt*-farnesol on cariogenic bacteria, which it has been studied for nearly 2 decades. So far the literature has been consistent in demonstrating the effect of *tt-farnesol* on the reduced production of EIP by Gtfs of *S. mutans* biofilms in both *in vitro* and *in vivo*, which is significantly important since it is well established the critical role of EIP in the adherence and colonization of this specific cariogenic microorganism on tooth surfaces (Schilling and Bowen, 1992; Yamashita et al., 1993) showing a direct impact on the initiation and progression of a carious lesion. The action of *tt*-farnesol on important virulence factors such as EIP production and amount of viable cells at later stages (3 and 5 days) of biofilm formation could be because the antibacterial agent got trapped within the polymeric chain and was being slowly released onto the medium, thus not having immediate contact with the cariogenic biofilm. This mechanism of action has been previously reported in the literature; for example, the commercial product Gluma 2 Bond that contains glutaraldehyde has been demonstrated that requires at least 24 h. to produce any antibacterial effect against *S. mutans* (André et al., 2015). In the same study, chlorhexidine-containing adhesive Peak Universal Bond killed only strict anaerobic microorganisms also after 24 h. It was previously demonstrated the effect of the antibacterial properties and dentin bond strength of experimental adhesives formed with the addition of *tt*-farnesol into 2 commercial products, Clearfil S3 Bond Plus which is a 7th generation (single step self-etch) and OptiBond S, a 5th generation (two-step etch-and-rinse) adhesive systems (André et al., 2017). In the mentioned study, there was no reduction of bacterial viability measured in CFU for any of the experimental adhesives tested. Also, the experimental adhesive formed with OptiBond S + *tt*-farnesol showed significant reduction in EIPs production that is in agreement with our results. Meanwhile in the mentioned study there was not a significant reduction in dentin bond strength for the experimental composites formed with the 2 commercial composites and *tt*-farnesol, in our study we found a significant reduction in dentin bond strength that seems to be associated with the reduction DC values. Recently, it was reported a universal adhesive incorporated with epigallocatechin-3-gallate, which is a polyphenol present in green tea and is known to have antioxidant, antimicrobial, anti-diabetic, and anti-inflammatory properties (Silva et al., 2020). Here it was demonstrated the antimicrobial potential of an experimental adhesive at 0.5% concentration of epigallocatechin-3-gallate against *S. mutans* biofilm, however, it showed significantly higher water sorption and solubility.

Dental adhesives are used to bond dental restorations to the tooth and have a direct contact with the dental substrate, which makes it relevant to investigate in whether the incorporation of an antibacterial agent (such as *tt*-farnesol) would help reduce the development of secondary caries. Nevertheless, it is essential to also evaluate if the incorporation of *tt*-farnesol has any effect on the intrinsic chemical properties as well as the bonding and sealing capabilities of the adhesive system. In this study the pH, DC, μTBS, and hybrid layer permeability were evaluated. The adhesive system used in this study is the so called “Universal” or “multi-mode” and represents the latest generation of dentin bonding system launched in the market. Is designed to be used in either one-step self-etch or in a two-step etch-and-rinse mode (Hanabusa et al., 2012; Wagner et al., 2014) without compromising the material’s bonding effectiveness (Muñoz et al., 2013). Additionally, universal adhesives also include in their formulation certain components that allow to chemically bond to zirconia or silica-based glass ceramics without the application of accessory priming agents (Özcan and Bernasconi, 2015; Siqueira et al., 2016). The pH or “acidity” of the adhesive system is strongly correlated to the material’s ability to demineralize dentin and enamel that result in varying etching depths (Van Meerbeek et al., 2011). Therefore, it important that added compounds such as *tt*-farnesol do not modify the material’s pH and its ability to etch the dental substrate. The pH evaluation revealed that all experimental adhesives with different concentrations of *tt*-farnesol yielded similar pH values (*P* > 0.05) to that of the control (pH ≈ 3) demonstrating that the intrinsic acidity of the material remained unaltered.

Degree of conversion is a quantitative evaluation of the monomer conversion after polymerization of light-cured dental materials, which may predict the bonding and mechanical capabilities of the system (Dickens and Cho, 2005; Kanehira et al., 2006). Results of this study reveal that the addition of *tt*-farnesol in different concentrations resulted in a significant reduction (*P* < 0.05) ranging from 32 to 37% decrease of reacted monomers of the experimental adhesives, which could compromise the adhesive bonding and sealing ability of the material. It is known that the incomplete polymerization of dental adhesive systems can leave a fraction of residual monomers that can diffuse through the dentinal tubules reaching the pulp tissue or leach into the neighboring soft tissues resulting in adverse reactions. Nonetheless, the result of a recent systematic review demonstrates that there is still a lack of agreement amongst the literature regarding the relationship between cytotoxicity and DC of dental adhesive systems (Caldas et al., 2019). Further analysis of the cytotoxic effects of leached monomers of experimental composites containing *tt*-farnesol is needed to ensure the safe use of this adhesive system in the oral environment. The bonding capability of the experimental adhesives to human dentin in both application modes: one-step self-etch and two-step etch-and-rinse was then evaluated. μTBS test to dentin produced values that ranged between 64.29 and 55.59 MPa in the etch-and-rinse mode, and values in the self-etch mode that ranged between 66.90 and 57.69 MPa of the experimental adhesives used in this study. There was a significant reduction (*P* < 0.05) in the bond strength in the experimental adhesive systems irrespective of the application mode when compared to its control. These results appear to be similar to the DC results, suggesting a relationship between decreased DC values and reduced bonding effectiveness of the experimental composites. When comparing both application modes, the observed bonding properties of the experimental composites evaluated in this study go somewhat in accordance with results observed in the current literature, that dictates that the adhesive performance of universal adhesives such as Adper Scothbond Universal is not dependent on the application mode used, suggesting that they can be used in both etch-and-rinse or self-etch mode. The use of previous step of phosphoric acid before the adhesive application has shown to remove the smear layer and smear plugs facilitating the penetration of the adhesive and the generation of longer resin tags (Langer and Ilie, 2013). On the other hand, in self-etch mode, the success of the bonding capability relies on partial demineralization of dentin and chemical bonding to hydroxyapatite (HAp) produced by the action of functional monomers such as 10-MDP present in the adhesive system (Wagner et al., 2014).

Confocal Laser Scanning Microscopy images illustrate the integration of the adhesive system and the dentinal fluids, allowing a better understanding of the permeability of the experimental adhesives into dentin. In the etch-and-rinse application mode, similar hybrid layer and resin tag formation was observed in all tested groups (Figure 6A). It can be noted that groups 1.90% (v/v) and 3.80% (v/v) displayed small droplets of unpolymerized monomers that leached out from the resin tags within the dentinal tubules, indicative of a reduced monomer conversion. These droplets are formed due to a phase separation of inadequate polymerization monomers of the experimental adhesive systems (Sartori et al., 2015). When the adhesive system was applied in the self-etch mode (Figure 6B) all adhesive systems (control and experimental) partially dissolved the smear layer and chemically bonded to dentin substrate to create a thin hybrid layer. However, the hybrid layer created by both the control and experimental groups demonstrated its inadequacy in properly sealing dentinal tubules from water permeation. In other words, all tested groups applied in the self-etch mode produced a semipermeable hybrid layer after polymerization (Sartori et al., 2016). The presence of pre-testing failures for the experimental adhesives used the in self-etch mode could be the result of neutralization of its etching capability to dentin; whereas, there were no pre-testing failures in the experimental adhesives when used in the etch-and-rinse mode. It was observed an evident increase in adhesive failures in both application modes at the highest concentration of *tt*-farnesol when compared to its control group. This can be explained because of the reduction in the mechanical properties as shown in the reduced monomer conversion that hindered the adhesion to the dental substrate of the adhesive systems.

In this study we observed the antibacterial effect of experimental adhesives containing *tt*-farnesol at concentrations that started at 0.38% (v/v), future work could be focused in evaluating lower concentrations of the antibacterial agent to possibly overcome the detrimental effects on the degree of monomer conversion and the resulting bonding effectiveness of the material without much compromise to the antibacterial properties. We evaluated the antibacterial effect on biofilms up to 5 days after surface treatment; this observation period could be extended to observe any differences between short-term and long-term effects also possibly including other important microorganisms that are present in mixed-species oral biofilms. Great effort is placed in the discovery and incorporation of antibacterial agents into dental materials; therefore, it would be relevant to introduce other natural agents that have shown important antibacterial properties against cariogenic microorganisms. For example, apigenin is another compound found in propolis that has shown to be an efficient inhibitor of Gtfs produced by *S. mutans* (Koo et al., 2002b). Another natural agent is 7-epiclusianone, a prenylated benzophenone isolated from the plant Rheedia gardneriana, has shown to greatly reduce DW, EIP, and the acidogenicity of *S. mutans* biofilms (Murata et al., 2008).

Although many factors are to account in the overall clinical performance and longevity of a restoration (Manhart et al., 2002), one of the main reasons for restoration failure is the development of secondary caries that demands the improvement of the current materials that are used for dental restorations. The creation of a dental adhesive system with antibacterial properties is a promising milestone in the advancement of preventive and adhesive dentistry; however, the complexity in developing an effective antibacterial adhesive is great. A better understanding of the antibacterial, chemical and bonding properties of experimental adhesives will guide the development of new bonding agents with antibacterial properties that overcome some limitations of current adhesive systems.

## Conclusion

The incorporation of *tt*-farnesol into a universal adhesive system reduced both the production of EIPs and the bacterial viability of *S. mutans* biofilm. However, the complex adhesive chemical composition created by the incorporation of antibacterial agent altered the original DC and bonding effectiveness of the adhesive system.

## Data Availability Statement

The datasets generated for this study are available on request to the corresponding author.

## Author Contributions

NS and RM contributed to the conception and design of the study. DL and NT carried sample preparation and experimentation. VP and NS contributed to the statistical analysis. J-HP, NS, RM, and SD contributed to the interpretation of the results. DL wrote the manuscript. All authors contributed to manuscript revision and approve the submitted version.

## Conflict of Interest

The authors declare that the research was conducted in the absence of any commercial or financial relationships that could be construed as a potential conflict of interest.

## Clinical Relevance

Adhesive systems with antibacterial properties might reduce the incidence of secondary caries adjoining to bonded interface and increase the lifespan of bonded restorations.
